# Investigation of the effect of cochlear implant electrode length on speech comprehension in quiet and noise compared with the results with users of electro-acoustic-stimulation, a retrospective analysis

**DOI:** 10.1371/journal.pone.0174900

**Published:** 2017-05-15

**Authors:** Andreas Büchner, Angelika Illg, Omid Majdani, Thomas Lenarz

**Affiliations:** Medical University Hannover, Dept. of Otorhinolaryngology, Germany; Universidad de Salamanca, SPAIN

## Abstract

**Objectives:**

This investigation evaluated the effect of cochlear implant (CI) electrode length on speech comprehension in quiet and noise and compare the results with those of EAS users.

**Methodes:**

91 adults with some degree of residual hearing were implanted with a FLEX^20^, FLEX^24^, or FLEX^28^ electrode. Some subjects were postoperative electric-acoustic-stimulation (EAS) users; the other subjects were in the groups of electric stimulation-only (ES-only).

Speech perception was tested in quiet and noise at 3 and 6 months of ES or EAS use. Speech comprehension results were analyzed and correlated to electrode length.

**Results:**

While the FLEX^20^ ES and FLEX^24^ ES groups were still in their learning phase between the 3 to 6 months interval, the FLEX^28^ ES group was already reaching a performance plateau at the three months appointment yielding remarkably high test scores. EAS subjects using FLEX^20^ or FLEX^24^ electrodes outscored ES-only subjects with the same short electrodes on all 3 tests at each interval, reaching significance with FLEX^20^ ES and FLEX^24^ ES subjects on all 3 tests at the 3-months interval and on 2 tests at the 6- months interval. Amongst ES-only subjects at the 3- months interval, FLEX^28^ ES subjects significantly outscored FLEX^20^ ES subjects on all 3 tests and the FLEX^24^ ES subjects on 2 tests. At the-6 months interval, FLEX^28^ ES subjects still exceeded the other ES-only subjects although the difference did not reach significance.

**Conclusions:**

Among ES-only users, the FLEX^28^ ES users had the best speech comprehension scores, at the 3- months appointment and tendentially at the 6 months appointment. EAS users showed significantly better speech comprehension results compared to ES-only users with the same short electrodes.

## Introduction

Many people with severe or severe-profound hearing loss in the high frequencies have functional residual hearing in the low frequencies. For such people, electric-acoustic-stimulation (EAS) or hybrid systems, which combine the use of CI (electric stimulation, ES) and a hearing aid (acoustic stimulation) into one device, are advisable and can significantly benefit users, especially in difficult listening environments [1, 2, 3, 4, 5, 6]. For EAS candidates and for CI candidates with less residual hearing, CI manufacturers developed thin and straight electrodes with lengths between 16 mm and 31 mm. The aim of these developments was to preserve residual hearing, even when it is marginal, by minimizing intraoperative damage to the sensitive intracochlear structures; while at the same time offering good speech understanding with electrical hearing only. If a CI recipient does lose residual hearing due to surgery, his/her electrically stimulated hearing must be superior to his/her preoperative speech understanding results or he/she will not have derived benefit from implantation.

With electrodes of different lengths, different insertion depths can be achieved. Insertion depth is determined by both the length of the selected electrode and the length of each individual candidate’s cochlear duct. Studies have shown the impact of cochlea size on insertion depth [7, 8, 9, 10]. In [11] a correlation between the preservation rate of residual hearing and corresponding insertion angle of the electrode was found whereas further studies demonstrated that hearing preservation is also possible with long electrodes [12,13,14,15]. The insertion depth angles differ. The FLEX electrode array in 10 temporal bone specimens vary between 341°±22° for FLEX^20^, 477°±36° for FLEX^24^, and 587°±42° for FLEX^28^ [8], indicating significant differences between the electrode arrays with respect to the cochlear coverage.

The influence of insertion depth on performance with electrical stimulation was shown by Hamzavi & Arnoldner [16] and by Buchman et al. [17]. Although their results showed that not all candidates benefited from the maximum number of activated channels, Hamzavi & Arnoldner [16] recommend deep insertion in candidates with profound hearing loss. Buchman et al. [17] compared a STANDARD (31 mm) with a MEDIUM (24 mm) electrode array in a prospective randomized study with 7 subjects receiving the long and 6 subjects receiving the shorter electrode array and found that deeper electrode insertion and greater insertion angles appear to offer better speech perception performance in the early post-activation period. It is interesting to mention that an interim analysis of the data in the latter study led to a discontinuation of the subject enrollment by the institutional review board because of the trend for better performance and faster improvement in the long electrode group. Further studies have found a positive correlation between the insertion depth and CI users’ speech perception [18, 19, 20, 21].

Other researchers found no positive or even negative correlation between insertion depth and speech understanding [22, 23, 24, 25, 26] with the caveat, however, that many different devices, electrode types and surgical techniques have been investigated and compared in these studies, introducing a high level of variability and complexity.

The aim of the present study was to analyze the influence of insertion depth and electrode length on the speech comprehension of ES-only and EAS users after 3- and 6-months of device use. The present study, with its comparatively large number of subjects, indicates that electrode length does play an important role in the ES-only user’s ability to hear in difficult listening environments. Almost all subjects evaluated in this study had residual acoustic hearing to some extent.

## Materials and methods

### Ethics statement

The Ethics committee of the Medical University of Hannover, Germany, approved this retrospective study. No written information was given to patients because of the retrospective design. All patient data were anonymized and de-identified prior the retrospective analysis.

### Subjects

The speech comprehension data of 91 cochlear implant patients were retrospectively analyzed after 3 and 6 months of device use. All subjects were postlingually hearing-impaired adult CI users who were implanted with a SONATA or CONCERTO with a FLEX^20^, FLEX^24^, or FLEX^28^ (MED-EL, Innsbruck, Austria) electrode array. Eighty five subjects were unilaterally implanted with FLEX electrodes. Sixty four of them used hearing aids on the contralateral side, 11 subjects were bilaterally implanted with standard electrodes by different CI companies on the contralateral side and 10 subjects used the implant unilaterally. Six subjects of this study were bilaterally implanted with FLEX electrodes, yielding a dataset of 97 ‘ears’. See Table 1 for subject demographics.

**Table 1:** Subject demographics.

	Age at study	Duration of hearing impairment	Duration of deafness	Age at implantation
All Patients Mean (SD) (range) in years	62.81 (14.44) (19–89)	23.2 (14.5) (2–84)	0.92 (2.43) (0–13)	62.76 (14.45) (19–89)
Group A- Mean (SD)	57.60 (12.09)	22.93 (10.35)	0 (0)	57.60 (12.09)
Group B- Mean (SD)	62.60 (17.30)	26.14 (20.42)	0.30 (1.11)	62.60 (17.30)
Group C- Mean (SD)	63.75 (13.50)	23.21 (12.54)	0.29 (0.99)	63.76 (13.55)
Group D- Mean (SD)	64.54 (14.09)	21.46 (13.08)	2.14 (3.58)	64.46 (14.01)

Etiology of deafness was unknown in 49 subjects, sudden hearing loss in 22 subjects, genetic in 7 subjects, viral in 4 subjects, noise-induced in 2 subjects, and 1 for each of the following: brainstem infarct, LVA-Syndrome, presbyacusis, otitis media, oxygen deficiency during birth, rubella embryopathy, and stapedectomy.

**Fig 1:**
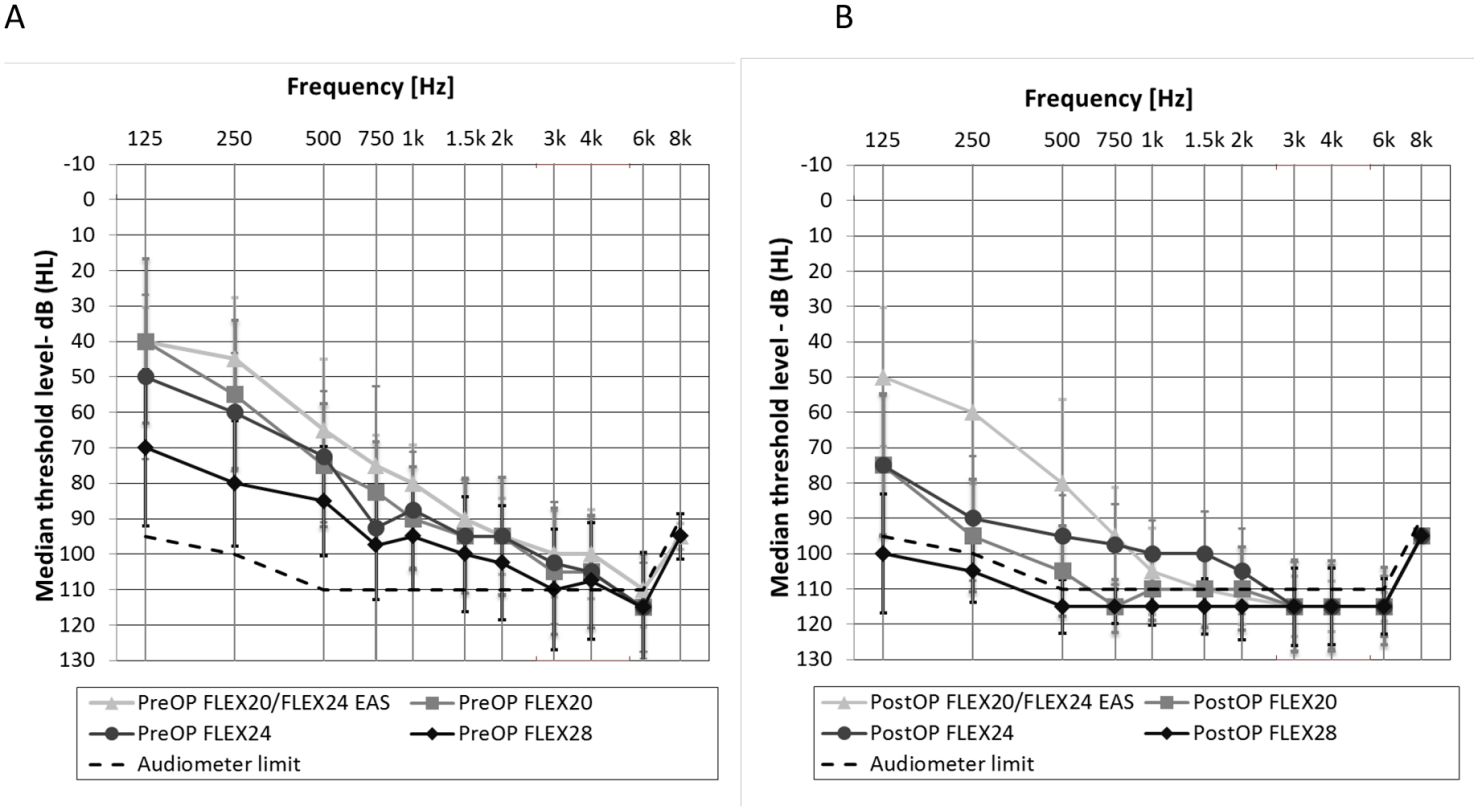
Pre- and postoperative median hearing levels, minimum and maximum for all 97 ears, divided into groups: A = preoperatively, B = postoperatively.

Subjects were divided into 4 groups depending on the length of their FLEX electrode and if they were an EAS or ES-only user. Group A consisted of EAS users with a FLEX^20^ or FLEX^24^ electrode; group B consisted of CI-only users with a FLEX^20^ electrode; group C consisted of CI-only users with a FLEX^24^ electrode; group D consisted of CI-only users with a FLEX^28^ electrode (Table 2). One subject switched from the EAS group (FLEX^24^ EAS) to the FLEX^24^ ES-only group after 3 months because he/she had insufficient residual hearing to benefit from continued acoustic amplification. Most subjects had some degree of pre- and postoperative residual hearing (Fig 1). In the subgroup of six patients which were bilaterally included in the study four patients were assigned with both ears to the same group: three patients in group A (FLEX^20^ EAS and FLEX^24^ EAS) and one in group D (FLEX^28^ ES). For one patient one ear was assigned to group C (FLEX^24^ ES) and the other ear in group D and for one patient one ear was assigned to group A and the other ear to group C and the patient switched to 6—months with both ears to group C. As the ears of the bilaterally included patients were tested independently, their results were treated as independent measurements.

**Table 2:** Subjects by group and their differences.

Group	Electrode	Length	N = 3 month	N = 6 month	EAS or ES	Audio processor	PTA pre-op 125–1000 Hz	PTA post-op 125–1000 Hz
A FLEX 20/24 EAS	FLEX^20^ or FLEX^24^	20 or 24 mm	15	14	EAS	DUET 2	56.69	76.80
B FLEX 20 ES	FLEX^20^	20 mm	23	23	ES	OPUS 2	70.34	96.42
C FLEX 24 ES	FLEX^24^	24 mm	24	25	ES	OPUS 2	73.64	92.00
D FLEX 28 ES	FLEX^28^	28 mm	35	35	ES	OPUS 2	85.59	104.2

Patients were assigned to the electrode array based on their pre-operatively residual hearing. If patients had significant residual hearing in the low frequencies, they could choose to receive short electrode types, i.e. FLEX^20^ or FLEX ^24^, because of the possibility to use EAS postoperatively.

The postoperative residual hearing was measured about 6 weeks after implantation during the first fitting phase. For documentation of the residual hearing, the air-conduction hearing loss was measured at single frequencies between 125 Hz and 8 kHz. If no hearing could be measured up to the measurement limit of our audiometer, the threshold was set to audiometer limit + 5 dB. This is a best case assumption and corresponds to the audiometer limit + the minimum step size of the audiometer. The option for an electric-acoustic simulation was offered to all patients with a residual hearing of at least 75 dB at 250 Hz at the time of first fitting. If subjects had less residual hearing or did decide not to use acoustic amplification they were allocated to one of the ES-user groups (B, C, or D). The subjects in groups B, C, and D (ES user) had a variety of reasons for not using any acoustic amplification in everyday life (i.e. not being EAS users). Some had insufficient residual hearing postoperatively to use acoustic amplification, one did not like having the ear mold in his/her auricle, and one complained about tinnitus, others had no subjective benefit when wearing the acoustic stimulation. Some FLEX^20^ and FLEX^24^ subjects were not typical EAS-candidates, but asked for full preservation of their residual hearing, even though it was very little. For that reason, these subjects received either the FLEX^20^ or FLEX^24^ electrode—depending on how precious their residual hearing was to them, with the assumption that the shorter array gives better preservation than the longer one.

### Fitting

In groups (B-D) with ES-only stimulation, 80 of the 82 ears used fine structure strategies FSP (fine structure processing) or FS4 since the time of first fitting. Only one patient of group D (bilaterally included in this study) was fitted with HDCIS (high-definition continuous interleaved sampling).

Both strategies are based on the CIS (continuous interleaved sampling) strategy described in [27]. The CIS strategy extracts the envelope information of each frequency band and provides a proportional stimulation current to the electrode contacts. The most recent CIS variant implemented in the MED-EL system is called HDCIS. With the fine structure strategies, additional temporal information is provided in the low frequency channels. This is done by dynamically adapting the stimulation rate on the most apical channels depending on the incoming acoustic signal [28]. The older implementation FSP offers rate pitch information on up to three fine structure channels, whereas FS4 guarantees fine structure information on the four most apical channels always.

In the group (A) with electric-acoustic-stimulation, 14 of 15 ears used fine structure strategies FSP or FS4 since their first activation. Only one patient (unilaterally included in this study) was fitted with HDCIS.

The low-frequency boundaries were primarily set based on the patients’ postoperative residual hearing and the patients’ subjective feedback.

Initially, the crossover-frequency between electric and acoustic hearing was set at the audiometric frequency where the hearing loss exceeds 65 to 80 dB HL. Based on patient’s feedback it was further adjusted and the preferred low-frequency boundary chosen. The resulting low-frequency cut-offs are between 100 Hz and 600 Hz.

### Speech comprehension testing

After 3 and 6 months of device use, all subjects were tested using the Freiburg monosyllabic word test (FMT) [29] in quiet and the German language Hochmair-Desoyer, Schulz, Moser sentence test (HSM test) in quiet and in noise at 0° azimuth (S0N0) at a 10 dB signal-to-noise ratio (SNR) [30]. All tests were performed in free field with sentences and monosyllables presented at 65 dB SPL. The tests were conducted in the subjects’ everyday life condition with regard to their ipsilateral ear, meaning that the subjects in group A were tested in EAS-mode and with a closed contralateral ear.

The subjects in group B, C, and D were tested with open ipsilateral ear.

To avoid bilateral benefit, the CI or hearing aid of the contralateral side was turned off and taken away. Additionally, the contralateral ear was plugged in bimodal patients or bilateral EAS patients to eliminate the influence of the residual hearing.

Not every patient could complete each of the tests and subsequently, the number of subjects varied between tests. As a consequence, each figure contains the related number of patients for each condition.

### Statistical analysis

Because the data were not normally distributed, the Mann-Whitney U Test for independent samples was used to evaluate hearing thresholds, demographic factors and speech comprehension data between the 4 groups. To compare the speech data gathered at the 3 and 6 months intervals, the Wilcoxon signed-rank test for dependent samples was used. For this comparison, patients’ data were excluded if only one of the two data sets (i.e. 3 or 6 months) was available. To investigate if demographic factors contribute to the speech data results, a hierarchical multiple linear regression analysis was performed. Dummy variables were defined in order to include the group variables (group A- group D) as categorical predictors into the model. The probable confounding factors age of implantation and duration of hearing impairment were added as predictors to the model in a stepwise manner. Based on the distribution of the predictors vs. dependent variable for all predictor variables a linear model was assumed without transformation. In order to check the assumption of linearity and heteroscedasticity the standardized residuals were checked for normal-distribution and for random distribution along the standardized predicted values. Statistical significance was set to p<0.05 (*p<0.05, **p<0.01, ***p<0.001). All data were analyzed statistically using IBM SPSS Statistics, 22.

## Results

### Hearing thresholds

The preoperative residual hearing in the low-frequencies (125 Hz– 1000 Hz) in patients receiving FLEX^20^ and FLEX^24^ electrodes was significantly better than for patients who received a longer FLEX^28^ electrode (FLEX^20^ *to* FLEX^28^: *Mann–Whitney U = 187*.*5*, *n*_*1*_ = *36*, *n*_*2*_ = *35*, *p<0*.*001*, FLEX^24^ *to* FLEX^28^: *Mann–Whitney U = 205*.*5*, *n*_*1*_ = *16*, *n*_*2*_ = *35*, *p<0*.*001)*. Comparing the air-conduction hearing levels along the groups, patients with FLEX^28^ ES showed the poorest mean scores in the low frequencies PTA (125 Hz– 1000 Hz) preoperatively (Table 2). Significant differences were found between the residual hearing in the low frequencies of the EAS group (group A) compared to all single ES groups preoperatively *(group A to group B*: *Mann–Whitney U = 77*.*5*, *n*_*1*_ = *15*, *n*_*2*_ = *23*, *p = 0*.*005*, *group A to group C*: *Mann–Whitney U = 62*.*5*, *n*_*1*_ = *15*, *n*_*2*_ = *24*, *p = 0*.*001*, *group A to group D*: *Mann–Whitney U = 16*.*5*, *n*_*1*_ = *15*, *n*_*2*_ = *35*, *p<0*.*001)*. Between FLEX^20^ ES and FLEX^24^ ES no significant differences were found *(pre-op*: *Mann–Whitney U = 274*, *n*_*1*_ = *23*, *n*_*2*_ = *24*, *p = 0*.*966)* in residual hearing.

Postoperatively, significant differences were found between the residual hearing in the low frequencies of the EAS group (A) compared to all ES groups (B-D) *(group A to group B*: *Mann–Whitney U = 45*, *n*_*1*_ = *15*, *n*_*2*_ = *23*, *p<0*.*001*, *group A to group C*: *Mann–Whitney U = 67*, *n*_*1*_ = *15*, *n*_*2*_ = *21*, *p = 0*.*004*, *group A to group D*: *Mann–Whitney U = 14*, *n*_*1*_ = *15*, *n*_*2*_ = *35*, *p<0*.*001)*. Between FLEX^20^ ES (group B) and FLEX^24^ ES subjects (group C), no significant differences were found *(post-op*: *Mann–Whitney U = 182*.*5*, *n*_*1*_ = *23*, *n*_*2*_ = *21*, *p = 0*.*165)* in residual hearing. Group A (FLEX^20^ EAS and FLEX^24^ EAS) showed the best PTA (125 Hz– 1000 Hz) postoperatively (Table 2).

### Demographic factors

In the demographics (Table 1) no significant difference were found in age of implantation and duration of hearing impairment between the groups. In duration of deafness a significant difference was found between group D compared to all other groups *(group A to group D*: *Mann–Whitney U = 157*.*5*, *n*_*1*_ = *15*, *n*_*2*_ = *35*, *p = 0*.*05*, *group B to group D*: *Mann–Whitney U = 274*, *n*_*1*_ = *23*, *n*_*2*_ = *35*, *p = 0*.*09*, *group C to group D*: *Mann–Whitney U = 283*.*5*, *n*_*1*_ = *24*, *n*_*2*_ = *35*, *p = 0*.*07)*, because patients who had no residual hearing pre-operatively received a longer FLEX^28^ electrode and were assigned to group D, whereas in the groups A to C the duration of deafness was close to zero as the patients had still residual hearing. Between the groups A-C no significant difference we found in duration of deafness.

### Speech comprehension

#### 3- months interval

**Fig 2:**
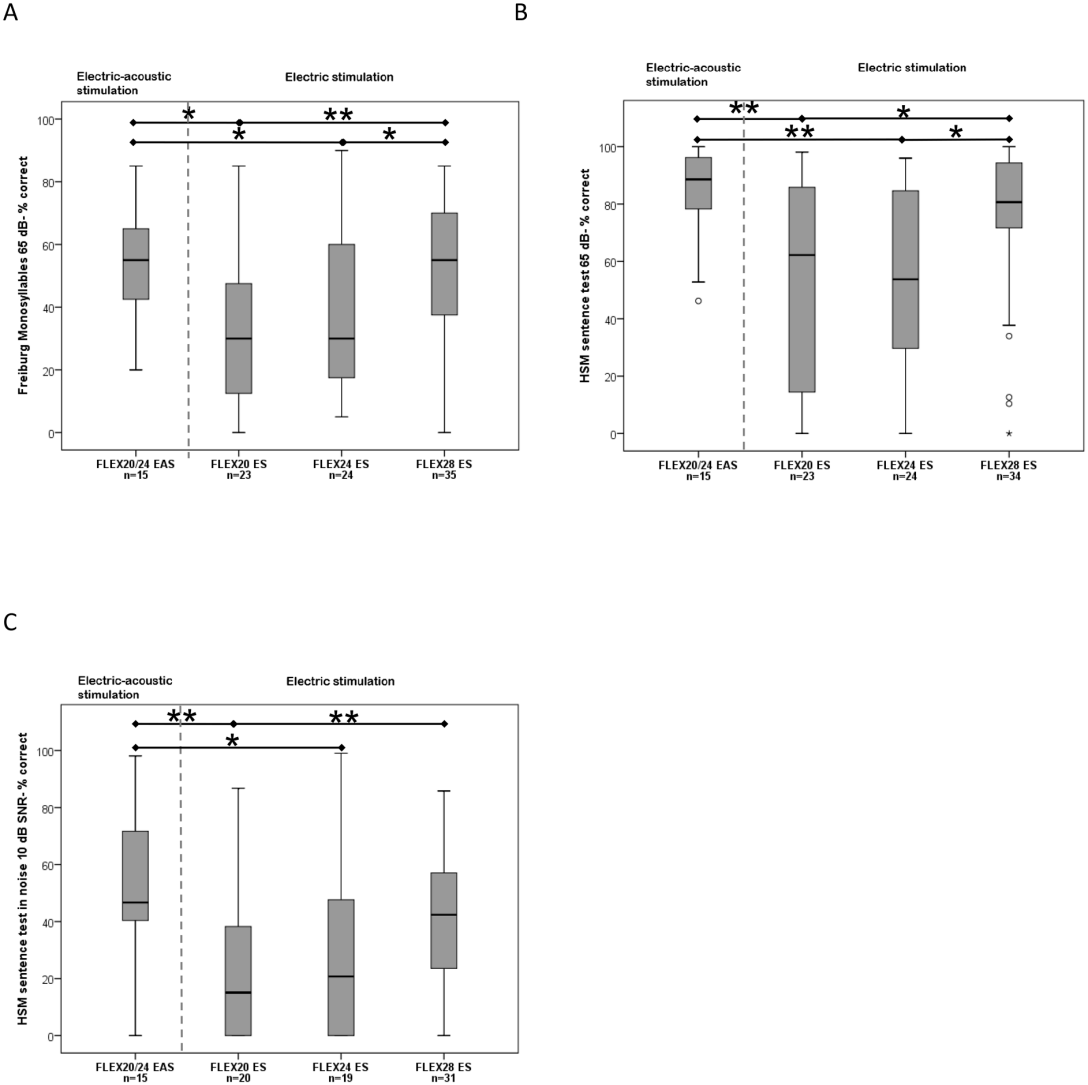
Median, interquartile, and minimum and maximum scores after 3 months, statistical significance is marked with * for p<0.05 and ** for p<0.01 A) Freiburg Monosyllables, B) HSM sentences in quiet, C) HSM sentences in noise.

1. FMT Scores: After 3 months of device use, groups B-D (ES-only) had the following mean scores: FLEX^20^ ES 35.11% (±24.29%), FLEX^24^ ES: 38.13% (±24.22%), and FLEX^28^ ES: 52.57% (±22.34%). The subjects using EAS scored a mean 55.17% (±18.72%). The median for EAS users was: 55.0%. For the ES-only groups the median was: FLEX^20^ ES: 30.0%, FLEX^24^ ES: 30.0%, and FLEX^28^ ES: 55.0% (Fig 2A). EAS subjects scored significantly better than FLEX^20^ ES users *(Mann–Whitney U = 87*.*5*, *n*_*1*_ = *15 n*_*2*_ = *23*, *p = 0*.*011)* and FLEX^24^ ES users *(Mann–Whitney U = 103*, *n*_*1*_ = *15 n*_*2*_ = *24*, *p = 0*.*026)*. FLEX^28^ ES users scored significantly better than FLEX^20^ ES users *(Mann–Whitney U = 235*, *n*_*1*_ = *23 n*_*2*_ = *35*, *p = 0*.*008)* and FLEX^24^ ES users *(Mann–Whitney U = 280*.*5*, *n*_*1*_ = *24 n*_*2*_ = *35*, *p = 0*.*031)*. The scores of the EAS subjects and FLEX^28^ ES users did not show significant differences.

2. HSM in quiet: After 3 months of device use, groups B-D (ES-only) had the following mean scores: FLEX^20^ ES: 51.98% (±37.22%), FLEX^24^ ES: 54.58% (±31.47%), and FLEX^28^ ES: 74.86% (±26.36%). The subjects using EAS scored a mean 83.87% (±16.96%). The median for EAS users was: 88.6%. For the ES-only groups the median was: FLEX^20^ ES: 62.2%, FLEX^24^ ES: 53.77%, and FLEX^28^ ES: 80.66% (Fig 2B). EAS users scored significantly better than FLEX^20^ ES users *(Mann–Whitney U = 79*, *n*_*1*_ = *15 n*_*2*_ = *23*, *p = 0*.*05)* and FLEX^24^ ES users *(Mann–Whitney*: *U = 80*, *n*_*1*_ = *15 n*_*2*_ = *24*, *p = 0*.*004)*. FLEX^28^ ES users scored significantly better than FLEX^20^ ES users *(Mann–Whitney U = 255*, *n*_*1*_ = *23 n*_*2*_ = *34*, *p = 0*.*027)* and FLEX^24^ ES users *(Mann–Whitney U = 256*.*5*, *n*_*1*_ = *24 n*_*2*_ = *34*, *p = 0*.*017)*. The scores of the EAS subjects and FLEX^28^ ES users did not show significant differences.

3. HSM in noise: After 3 months of device use, groups B-D (ES-only) had the following mean scores: FLEX^20^ ES: 21.13% (±25.00%), FLEX^24^ ES: 28.97% (±29.76%), and FLEX^28^ ES: 41.51% (±24.71%). The subjects using EAS scored a mean 50.50% (±26.52%). The median for EAS users was: 46.7%. For the ES-only groups the median was: FLEX^20^ ES: 15.09%, FLEX^24^ ES: 20.75%, and FLEX^28^ ES: 42.4% (Fig 2C). EAS users scored significantly better than FLEX^20^ ES users *(Mann–Whitney U = 60*.*5*, *n*_*1*_ = *15 n*_*2*_ = *20*, *p = 0*.*003)* and FLEX^24^ ES users *(Mann–Whitney*: *U = 81*, *n*_*1*_ = *15 n*_*2*_ = *19*, *p = 0*.*033)*. FLEX^28^ ES users scored significantly better than FLEX^20^ ES users *(Mann–Whitney U = 162*, *n*_*1*_ = *20 n*_*2*_ = *31*, *p = 0*.*004)*. The scores of the EAS subjects and FLEX^28^ ES users did not show significant differences.

#### 6- months interval

**Fig 3:**
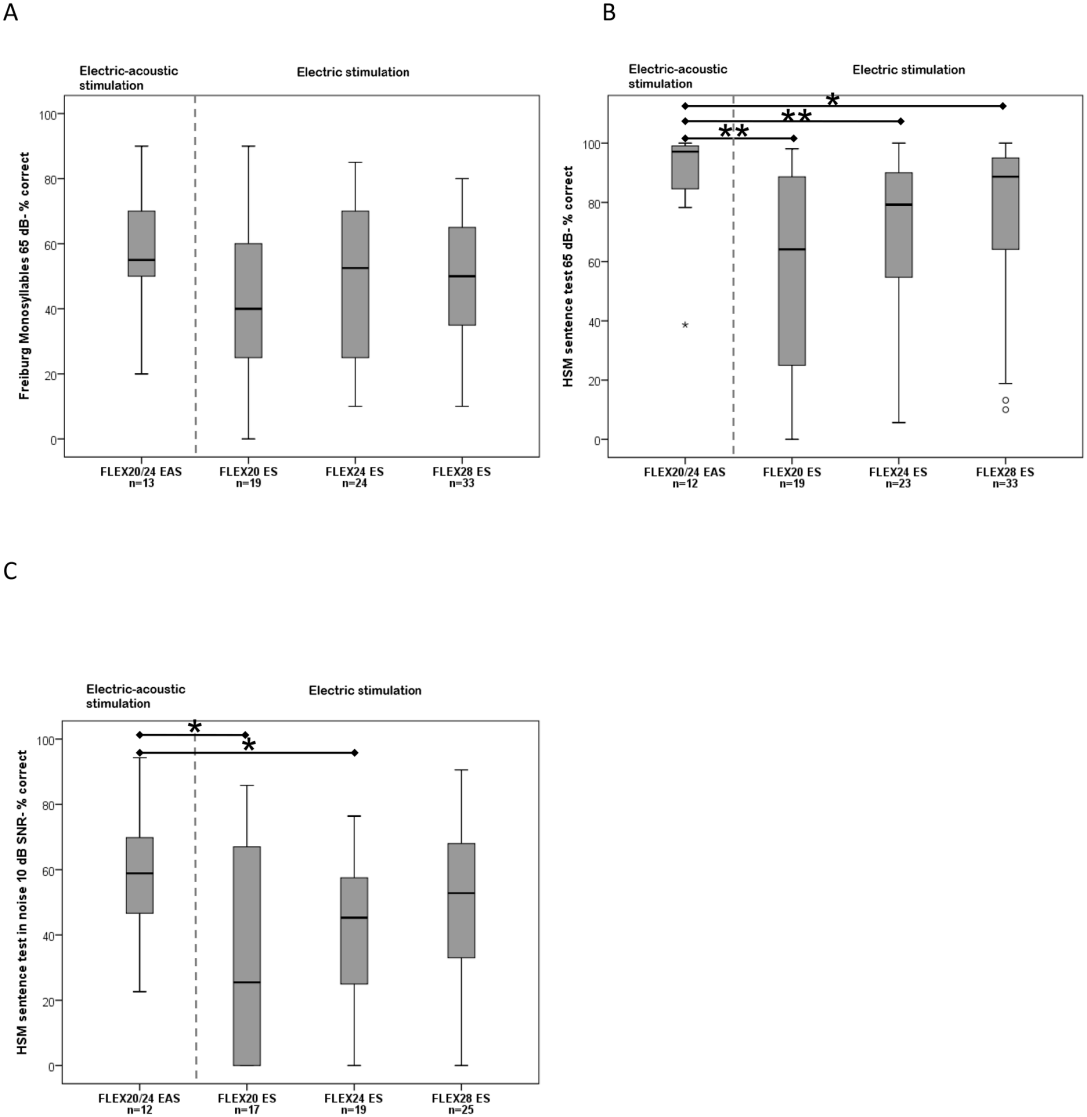
Median, interquartile, and minimum and maximum scores after 6 months: A) Freiburg Monosyllables, B) HSM sentences in quiet, C) HSM sentences in noise, statistical significance is marked with * for p<0.05 and ** for p<0.01.

1. FMT Scores: After 6 months of device use, groups B-D (ES-only) had the following mean scores: FLEX^20^ ES: 42.63% (±23.78%), FLEX^24^ ES: 47.29% (±23.77%), and FLEX^28^ ES: 49.09% (±20.06%). The subjects using EAS scored a mean 56.92% (±19.74%). No significant differences were found between groups. The median for EAS users was: 55.0%. For the ES-only groups the median was: FLEX^20^ ES: 40.0%, FLEX^24^ ES: 52.5%, and FLEX^28^ ES: 50.0% (Fig 3A). The scores of the EAS subjects and FLEX^28^ ES users did not show significant differences.

2. HSM in quiet: After 6 months of device use, groups B-D (ES-only) had the following mean scores: FLEX^20^ ES: 57.86% (±34.00%), FLEX^24^ ES: 68.80% (±27.17%), and FLEX^28^ ES: 74.35% (±28.08%). The subjects using EAS scored a mean 88.80% (±17.55%). The median for EAS users was: 97.13%. For the ES-only groups the median was: FLEX^20^ ES: 64.15%, FLEX^24^ ES: 79.24%, and FLEX^28^ ES: 88.67% (Fig 3B). EAS users scored significantly better than FLEX^20^ ES users *(Mann–Whitney U = 41*, *n*_*1*_ = *12 n*_*2*_ = *19*, *p = 0*.*003)*, FLEX^24^ ES users *(Mann–Whitney U = 60*.*5*, *n*_*1*_ = *12*, *n*_*2*_ = *23*, *p = 0*.*007)*, and FLEX^28^ ES users *(Mann–Whitney U = 115*.*5*, *n*_*1*_ = *12*, *n*_*2*_ = *33*, *p = 0*.*034)*.

3. HSM in noise: After 6 months of device use, groups B-D (ES-only) had the following mean scores: FLEX^20^ ES: 32.96% (±32.78%), FLEX^24^ ES: 38.65% (±25.40%), and FLEX^28^ ES: 49.44% (±26.18%). The subjects using EAS scored a mean 59.75% (±19.9%). The median for EAS users was: 58.88%. For the ES-only groups the median was: FLEX^20^ ES: 25.47%, FLEX^24^ ES: 45.28%, and FLEX^28^ ES: 52.83% (Fig 3C). EAS users scored significantly better than FLEX^20^ ES users *(Mann–Whitney U = 56*, *n*_*1*_ = *12*, *n*_*2*_ = *17*, *p = 0*.*041)* and FLEX^24^ ES users *(Mann–Whitney U = 64*, *n*_*1*_ = *12*, *n*_*2*_ = *19*, *p = 0*.*042)*. The scores of the EAS subjects and FLEX^28^ ES users did not show significant differences.

#### Comparison of the 3- and 6- months results

**Fig 4:**
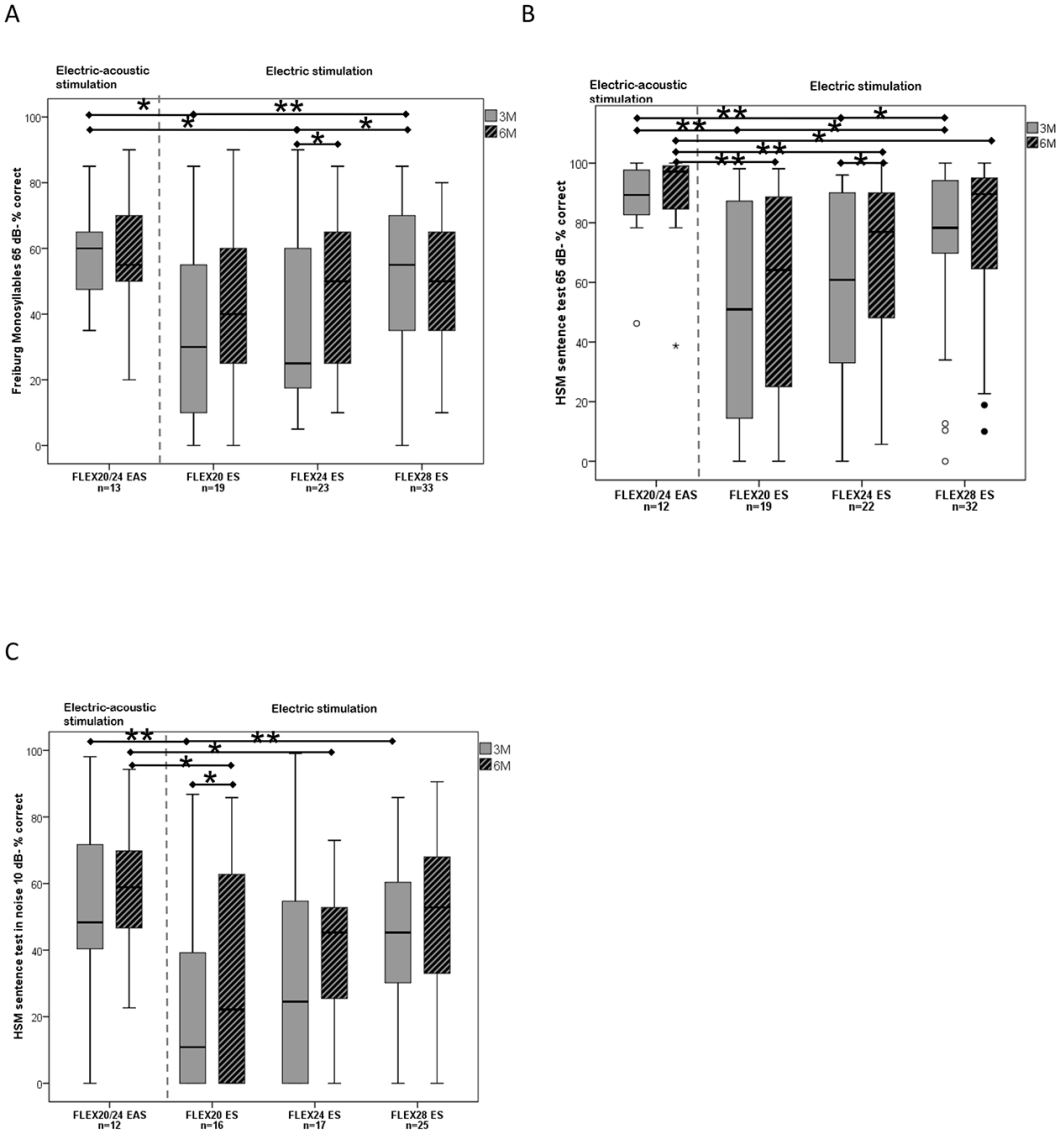
Median, interquartile, and minimum and maximum scores between scores after 3 and 6 months: A) Freiburg Monosyllables, B) HSM sentences in quiet, C) HSM sentences in noise. Significant differences of 3- and 6 months data and intragroup comparison are marked with * for p<0.05 and ** for p<0.01.

Intragroup comparison of the 3- and 6- months FMT results showed a significant difference only in the FLEX^24^ ES group, which had a significantly higher score at 6 months than at 3 months *(Wilcoxon signed-rank*: *T = 50*.*5*, *p = 0*.*023)* (Fig 4A).

Intragroup comparison of the 3- and 6- months HSM-sentence test in quiet scores showed a significant difference only in the FLEX^24^ ES group, which had a significantly higher score at 6 months than at 3 months *(Wilcoxon signed-rank*: *T = 32*.*5*, *p = 0*.*012*) (Fig 4B). FLEX^20^ ES and FLEX^24^ ES groups had a higher distribution of results than did the EAS and FLEX^28^ ES groups.

Intragroup comparison of the 3- and 6- months HSM-sentence test in noise scores showed a significant difference only in the FLEX^20^ ES group, which had a significantly higher score at 6 months than at 3 months *(Wilcoxon signed-rank*: *T = 32*.*5*, *p = 0*.*041*) (Fig 4C).

### Influence of demographic factors on the speech data

A hierarchical multiple regression analyses was performed in order to investigate the effect of demographic factors on the speech data and to determine if the group factor remains significant when including these confounding factors. Age of implantation and duration of hearing impairment were investigated as probable confounding factors. Exemplarily, the regression analysis was performed for the speech data of the FMT at 3 months. In the model the FMT was the dependent variable.

In the first step the regression analysis was performed with the group variables (A-D) as independent variables. Three dummy variables D1-D3 were defined for each ES group (group B- group D). The EAS group (group A) was defined as the baseline group and is specified when the three dummy variables are set to zero. Therefore, the effect of the dummy variables shows the effect of the ES groups in comparison to the EAS group.

Including the dummy variables has a significant effect on the model and explains 12.4% of the variance (*R*^*2*^ = *0*.*124*, *F (3*, *92) = 4*.*32*, *p = 0*.*007*). The model constant *b*_*0*,_ is 55.17 and corresponds to the mean value of the FMT score of group A. The model coefficients *b*, which are the slopes of the linear model, and the standardized coefficients *β* for the dummy variables are: D1: group B vs. group A: *b* = -20.05, *β* = -0.35 *(t = -2*.*61*, *p = 0*.*010*); D2: group C vs. group A: *b* = -17.04, *β* = -0.31 *(t = -2*.*26*, *p = 0*.*026*); D3: group D vs. group A: *b* = -2.60, *β* = -0.052 *(t = -0*.*37*, *p = 0*.*715*)

This shows that entering of D1 and D2 has a negative significant effect on the speech score. It means that the speech scores for group B and group C are significantly worse than those of group A. For Group D, no significant change compared to group A was found. This is in line with the findings of comparing the group medians.

When adding age of implantation as a predictor, the model lead to a significant increase in the prediction of variability (*F*_*change*_ *(1*, *91) = 8*.*23*, *p = 0*.*005*), and the overall model explains 19.6% of the variance *(R*^*2*^ = *0*.*196*, *F (4*, *91) = 5*.*56*, *p <0*.*001)*. The model constant *b*_*0*_ changes to 81.44. The model coefficients *b* and the standardized coefficients *β* for age of implantation are *b* = -0.46, *β* = -2.73 *(t = -2*.*87*, *p = 0*.*005*), and change for the dummy variables to: D1: group B vs. group A: *b* = -17.45, *β* = -0.31 *(t = -2*.*34*, *p = 0*.*021*); D2: group C vs. group A: *b* = -14.28, *β* = -0.26 *(t = -1*.*95*, *p = 0*.*055*); D3: group D vs. group A: *b* = 0.53, *β* = 0.11 *(t = 0*.*77*, *p = 0*.*939*).

This shows that age of implantation has a negative significant effect on the speech scores, whereas the effect of dummy variable D1 stays significant and D2 still shows a clear trend if including age of implantation into the model. This means that the effect of the groups is slightly decreased when adding age of implantation, but the group variables still explain most of the model variance.

Adding duration of hearing impairment as a predictor did not show an additional significant effect on the model (*F*_*change*_ *(1*, *91) = 8*.*23*, *p = 0*.*005*). It explains 19.9% of the variance *(R*^*2*^ = *0*.*199*, *F (4*, *91) = 4*.*49*, *p = 0*.*001)*. The model constant *b*_*0*_ changes to 83.55. The model coefficients *b* and the standardized coefficients *β* are *b* = -0.90, *β* = -0.54 *(t = -0*.*57*, *p = 0*.*572*) for duration of hearing impairment and *b* = -0.46, *β* = -2.74 *(t = -2*.*87*, *p = 0*.*005*) for age of implantation, and they change for the dummy variables to D1: group B vs. group A: *b* = -17.15, *β* = -0.30 *(t = -2*.*29*, *p = 0*.*024*); D2: group C vs. group A: *b* = -14.24, *β* = -0.26 *(t = -1*.*94*, *p = 0*.*056*); D3: group D vs. group A: *b* = 0.41, *β* = 0.08 *(t = 0*.*59*, *p = 0*.*953*).

This means that duration of hearing impairment does not affect the speech scores and does not change the effect of the group variables and of age of implantation on the speech scores.

## Discussion

We analyzed the speech understanding outcomes of subjects who had undergone residual hearing preservation CI surgery using MED-EL implants with different lengths of FLEX electrodes. In 2006, Hamzavi & Arnoldner [16] showed by deactivating apical electrodes in subjects implanted with the 31.5 mm length standard MED-EL electrode that deep stimulation of the cochlea enhances speech understanding. However, as the different conditions in this particular study were tested acutely without time for adaptation, the results are of limited significance only. Buchman et al. [17], who examined speech understanding in subjects with MED-EL STANDARD (length 31.5 mm) or MEDIUM (length 24 mm) electrodes, showed that insertion with the longer electrode offered better speech perception performance, at least in the early postactivation period. However, subjective measurements like quality of life and music perception showed no difference between the cohorts. The authors suspected that deeper insertion angles with the standard arrays produce greater degrees of cochlear coverage in the apical regions and a better or more natural tonotopic place representation during stimulation. In this context, Landsberger et. al [31] evaluated data from 92 cochlear implantations and found that longer electrode arrays, in particular the MED-EL STANDARD and FLEX^28^, demonstrated much smaller deviations from the spiral ganglion map [32] than other electrode arrays. Our study also demonstrates better hearing results with longer electrode arrays—in particular the FLEX28—in the ES-only user group, which might in fact be attributable to the more natural frequency mapping. This hypothesis is supported by very recent data from Rader et al., who found more accurate pitch perception in cochlear implant subjects when the place of stimulation and the stimulation rate had been matched [33]. Also, there seems to be growing evidence that the apical region of the cochlear is more suitable for conveying adequate rate pitch for frequencies up to 300 Hz, thus giving patients with longer arrays and appropriate coding strategies access to low frequency information which is unavailable to short electrode subjects. Some studies [34, 35] suggested that temporal processing near the apex may be different compared to more basal locations in the cochlear. However, insertion depth has to be considered carefully on a case by case basis and overly deep insertions of short electrode arrays should be avoided, as there is evidence that the more basal parts of the cochlear still seem to be more relevant for speech understanding than the apical regions [36, 25, 26].

Another plausible explanation for better hearing in the subjects with longer arrays simply might be the larger electrode contact spacing with those electrodes leading to less crosstalk or channel interaction between adjacent contacts. There is evidence that less channel interaction leads to improved spectral resolution and better speech understanding in noise [37, 38, 39].

Our conclusions on this topic are obviously based on the data from the three groups with no usable acoustic hearing, namely FLEX^28^ ES, FLEX^24^ ES and FLEX^20^ ES. Although only 3- and 6- months data are available so far, it is apparent that greater insertion angles do significantly enhance speech understanding in these ES-only users, especially at the 3- months interval. Even at the 6- months appointment, subjects with the FLEX^28^ ES tendentially outperformed the ES-only FLEX^20^ ES and FLEX^24^ ES users, most notably in the two HSM tests, although the score differences did not reach significance. Also, the performance variation among the subjects was much smaller in the FLEX^28^ ES group than in the other two ES-only groups (Figs 3 and 4). From our clinical routine data, there is empirical evidence that ES-only subjects with conventional electrodes outperform ES-only subjects with a short electrode array (i.e. 20 mm and shorter). Such pronounced differences as seen in our current analysis were, however, not expected, partly because of the suggestion of a possible accommodation to shorter electrode lengths leading to comparable hearing performance after a few months [6]. Although the scores of the FLEX^20^ ES and FLEX^24^ ES groups were catching up to that of the FLEX^28^ ES group at 6 months after surgery, a noticeable difference nonetheless remained. It could be speculated that, although brain plasticity can compensate for the pitch-mismatch with short electrode arrays over time to a great extent, there are still limits to what it can actually achieve. In this context it is worth mentioning recent work by Reiss et al. [40], who demonstrated in subjects with residual acoustic hearing in the contralateral ear that the pitch fusion of the electric stimulus and an acoustic tone gets broader with increasing pitch-mismatch between the acoustic and electric modality, suggesting that brain plasticity—although apparently existing—has its limitations. Further observation of the study subjects would be very interesting to see if the FLEX^20^ ES and FLEX^24^ ES groups would eventually fully catch up with the FLEX^28^ ES group. In this context, it would also be interesting to include measures for listening-effort into the test battery, something which is currently being discussed at our center.

Subjects implanted with the shorter arrays had higher degrees of acoustic hearing prior to surgery, which was why they had been implanted with shorter electrode types (Fig 1A). Still, 3 months after initial switch-on, the HSM in noise test scores of subjects with the FLEX^20^ ES electrode who had lost usable residual hearing (i.e. the ES-only subjects with a FLEX^20^), were barely half of those of the FLEX^28^ ES group. It is also worth mentioning that the FLEX^28^ ES group (without acoustic hearing) and the EAS users group (with functional residual hearing) achieved very similar results on the HSM sentences in noise test. This is remarkable because EAS stimulation is generally considered far superior to electric stimulation only [41, 42]. However, when looking at subjects with comparable demographics, in particular preoperative residual hearing, the picture is not as clear: the advantage for the EAS subjects over the FLEX^28^ ES subjects is only marginal. One might even be tempted to play the devil’s advocate and argue that possible shortcomings of the shallow insertion of EAS electrodes are only just outweighed by the users being able to use their acoustic residual hearing. In other words: EAS stimulation might not give a huge benefit over conventional electric stimulation with longer electrodes, at least as reflected in speech test scores from tests conducted in artificial settings, i.e. in clinical testing. Of course, there is no substitute for natural acoustic hearing and EAS subjects still have advantages when it comes to listening to music [43, 44]. EAS subjects also seem to need to expend less effort when listening in multi-talker environments, where it is key to perceive high-fidelity low-frequency information in order to separate individual talkers from each other and to listen to the intended target speaker [41, 42, 43, 45, 46]. Furthermore, bimodal hearing subjects also usually show measurable improvements over hearing with a CI alone [47, 48, 49, 50]. On the other hand, in the bimodal group, the effect of listening with two input channels, i.e. two ears, one supplied with a hearing aid and one with a conventional CI, also needs to be taken into account as a possible reason for improved hearing scores in the bimodal condition, rather than simply the combination of electric and acoustic hearing alone. However, the current dataset shows the dilemma that subjects and clinicians are currently in: on one hand, residual hearing should be preserved to the highest possible extent and today this is only possible with comparatively short electrode arrays; on the other hand, when residual hearing is lost, either through surgery or over time, CI users with short arrays seem to be limited in their hearing capabilities compared to CI users with longer electrodes. An investigation into EAS hearing in subjects implanted with longer electrode arrays and good postoperative residual hearing would be highly useful in shedding light on this topic. This combination is rare, but occasionally we do have subjects who explicitly opt for the long electrode array despite having relatively good residual hearing. This group, however is too small at present to draw any conclusions. Also, it needs to be investigated if deeper insertion of longer electrodes, and with it the stimulation of more apical structures of the cochlea, is indeed the reason for the superior performance, or if the larger electrode spacing together with better channel separation is the key for the apparently better speech understanding of subjects implanted with longer electrode arrays.
